# Stability-Oriented Deep Learning for Hyperspectral Soil Organic Matter Estimation

**DOI:** 10.3390/s26020741

**Published:** 2026-01-22

**Authors:** Yun Deng, Yuxi Shi

**Affiliations:** 1 Guangxi Key Laboratory of Embedded Technology and Intelligent System, Guilin University of Technology, 12 Jiangan Road, Guilin 541004, China; 2002078@glut.edu.cn; 2 College of Computer Science and Engineering, Guilin University of Technology, 319 Yanshan Street, Yanshan District, Guilin 541006, China

**Keywords:** soil organic matter, hyperspectral sensing, data augmentation, deep learning, small-sample modeling

## Abstract

Soil organic matter (SOM) is a key indicator for evaluating soil fertility and ecological functions, and hyperspectral technology provides an effective means for its rapid and non-destructive estimation. However, in practical soil systems, the spectral response of SOM is often highly covariant with mineral composition, moisture conditions, and soil structural characteristics. Under small-sample conditions, hyperspectral SOM modeling results are usually highly sensitive to spectral preprocessing methods, sample perturbations, and model architecture and parameter configurations, leading to fluctuations in predictive performance across independent runs and thereby limiting model stability and practical applicability. To address these issues, this study proposes a multi-strategy collaborative deep learning modeling framework for small-sample conditions (SE-EDCNN-DA-LWGPSO). Under unified data partitioning and evaluation settings, the framework integrates spectral preprocessing, data augmentation based on sensor perturbation simulation, multi-scale dilated convolution feature extraction, an SE channel attention mechanism, and a linearly weighted generalized particle swarm optimization algorithm. Subtropical red soil samples from Guangxi were used as the study object. Samples were partitioned using the SPXY method, and multiple independent repeated experiments were conducted to evaluate the predictive performance and training consistency of the model under fixed validation conditions. The results indicate that the combination of Savitzky–Golay filtering and first-derivative transformation (SG–1DR) exhibits superior overall stability among various preprocessing schemes. In model structure comparison and ablation analysis, as dilated convolution, data augmentation, and channel attention mechanisms were progressively introduced, the fluctuations of prediction errors on the validation set gradually converged, and the performance dispersion among different independent runs was significantly reduced. Under ten independent repeated experiments, the final model achieved R^2^ = 0.938 ± 0.010, RMSE = 2.256 ± 0.176 g·kg^−1^, and RPD = 4.050 ± 0.305 on the validation set, demonstrating that the proposed framework has good modeling consistency and numerical stability under small-sample conditions.

## 1. Introduction

Visible–near infrared (Vis–NIR) hyperspectral technology, which provides continuous spectral information over the 350–2500 nm wavelength range, has been widely applied in agricultural and ecological environmental monitoring, enabling the characterization of multiple soil chemical and physical properties within a unified spectral framework. Hyperspectral sensors represented by the ASD FieldSpec series are capable of capturing reflectance variations related to soil organic matter (SOM), including absorption features, spectral slopes, and their coupled responses with mineral components. However, during practical measurements, hyperspectral data are susceptible to noise, illumination variability, and environmental scattering, introducing wavelength-dependent perturbations that increase the difficulty of quantitative SOM prediction. Especially when sample size is limited, extracting stable and discriminative features from high-dimensional spectral data remains a core challenge in SOM spectral modeling.

From the perspective of spectral mechanisms, soil reflectance characteristics in the Vis–NIR region are typically the integrated result of the combined effects of multiple soil constituents. In addition to the intrinsic absorption behavior of SOM, clay minerals, iron oxides, and the organic–mineral complexes they form also exert significant influences on spectral shape and absorption features [1]. In soil systems rich in iron oxides and clay minerals, such as red soils, variations in SOM content are often accompanied by coordinated changes in mineral-related spectral features. Therefore, hyperspectral SOM prediction places greater emphasis on effectively modeling the integrated spectral response patterns of specific soil systems, rather than directly decoupling the absorption features of individual chemical components.

As a key indicator for assessing soil fertility and ecosystem stability, SOM plays an important role in soil quality evaluation and ecological research [2]. Although traditional laboratory analytical methods offer high accuracy, their cost and efficiency make it difficult to meet the demands of rapid and large-scale monitoring [3]. With the development of hyperspectral sensing technologies, SOM prediction based on spectral data has gradually become an attractive alternative. Nevertheless, due to the nonlinear nature of spectral–soil relationships and variations in sensing conditions, constructing stable and reliable predictive models under small-sample conditions still faces significant challenges [4,5].

Regarding hyperspectral prediction of SOM, methods such as partial least squares regression (PLSR), random forest (RF), and support vector regression (SVR) have been widely applied [6,7,8,9]. In recent years, deep learning models, including convolutional neural networks (CNNs) and attention mechanisms, have further enhanced the capability to represent complex spectral features [10,11,12]. However, existing studies have indicated that under small-sample conditions, the prediction results of deep models are often highly sensitive to spectral preprocessing, network architecture, and training configurations, with uncertainty mainly manifested as performance fluctuations across different modeling settings and repeated training processes [13,14]. Although some studies have attempted optimization through data augmentation or network architecture improvements [15,16], systematic designs that consider the synergistic effects of multiple factors throughout the modeling pipeline remain relatively limited.

Based on the above background, this study proposes a multi-strategy collaborative deep learning modeling framework—SE-EDCNN-DA-LWGPSO—for hyperspectral soil organic matter prediction. The framework integrates spectral preprocessing, data augmentation strategies for simulating sensor perturbations, a multi-scale expanded dilated convolutional neural network (EDCNN), an SE channel attention mechanism, and a linearly weighted generalized particle swarm optimization algorithm (LWGPSO), aiming to achieve the synergistic optimization of model structure and hyperparameter configuration. The core focus of this study is not overfitting in the traditional sense, but rather the stability and consistency of model prediction results across different modeling configurations and repeated training processes under small-sample conditions. The proposed multi-strategy collaborative modeling concept has a certain degree of methodological generality and may provide reference for other hyperspectral regression studies constrained by sample size or sensor conditions.

## 2. Materials and Methods

### 2.1. Study Area

The study area is located in a state-owned forest farm in the Guangxi Zhuang Autonomous Region, China (22°49′–23°15′ N, 108°08′–108°53′ E). The region has a subtropical monsoon climate, characterized by abundant annual precipitation and stable vegetation cover. The forest farm covers an area of approximately 890,000 mu, with a forest coverage rate of 83.7% and a standing timber volume of about 7.0 million m^3^. The dominant soil type in the study area is subtropical red soil, with textures ranging from loam to light clay, exhibiting good water- and nutrient-holding capacities. These conditions are favorable for the accumulation of soil organic matter (SOM) and its spatial variability. The study area represents a typical subtropical red soil system and provides a representative application scenario for evaluating the modeling stability and predictive performance of hyperspectral SOM prediction methods under specific soil conditions.

### 2.2. Soil Sample Collection and Laboratory Measurements

**Figure 1:**
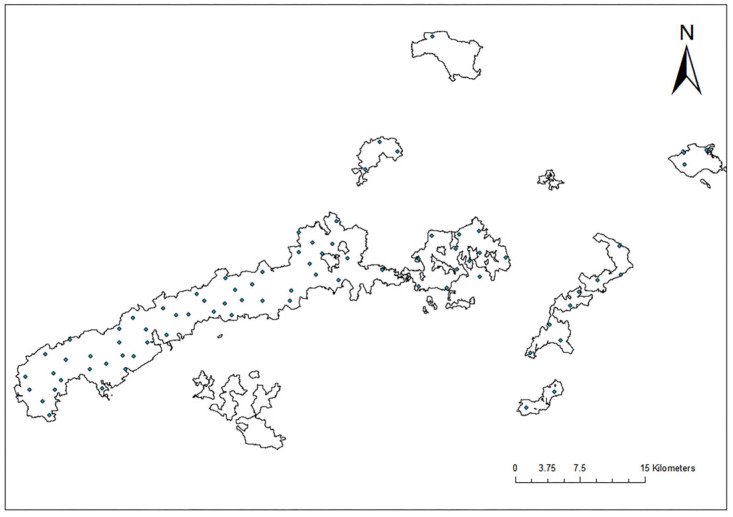
Spatial distribution of soil sampling points in the study area of Guangxi Province, China.

To obtain representative soil samples, a grid-based sampling strategy was adopted in this study, with a total of 88 sampling points established across the study area (Figure 1). Sampling was conducted prior to fertilization activities (March–April), and an S-shaped sampling pattern was used to collect surface soil from the 0–40 cm layer, yielding a total of 278 soil samples. This sampling depth was selected to characterize the integrated surface soil properties commonly considered in routine forest management and ecological assessments, rather than to distinguish spectral differences among individual pedogenic horizons.

Under laboratory conditions, all samples were air-dried, ground, and passed through a 2 mm sieve. Each sample was then divided into two portions: one was used for determining soil organic matter (SOM) content using the potassium dichromate oxidation method, and the other was used for hyperspectral reflectance measurements.

Hyperspectral reflectance measurements were performed using an ASD FieldSpec 4 Hi-Res spectroradiometer, which covers a spectral range of 350–2500 nm and is widely used in soil spectral studies. To ensure measurement stability, spectral acquisition was carried out against a dark background, with periodic white reference calibration using a Spectralon^®^ panel. Each soil sample was scanned ten times, and the averaged spectrum was used as the final result to reduce random noise and improve the signal-to-noise ratio (SNR).

### 2.3. Technical Framework

To address the issue that modeling results for hyperspectral soil organic matter (SOM) prediction under small-sample conditions are highly sensitive to data representation and model configuration and are prone to performance fluctuations, this study establishes a systematic technical framework consisting of spectral preprocessing, modeling, and evaluation. The overall workflow conducts a coordinated analysis and design of key factors affecting the stability and consistency of prediction results from the data level, feature learning level, and model configuration level.

In the spectral preprocessing stage, considering both noise suppression and feature enhancement requirements, 24 preprocessing combination schemes were designed. These include three denoising strategies (no denoising, Savitzky–Golay filtering, and Fourier transform filtering) and eight mathematical transformation forms (R, 1DR, 2DR, R1, R1D, RL, LNR, and LR), aiming to systematically evaluate the influence of different data representation methods on SOM modeling.

In the modeling stage, for each preprocessing combination, three traditional machine learning models (PLSR, RF, and SVR) and five deep learning models were constructed, including CNN, EDCNN, and their improved variants obtained by progressively introducing data augmentation (DA), an SE channel attention mechanism, and a linearly weighted generalized particle swarm optimization algorithm (LWGPSO). Through hierarchical model configurations, the predictive accuracy and result stability of different modeling strategies under small-sample conditions were comparatively analyzed.

**Figure 2:**
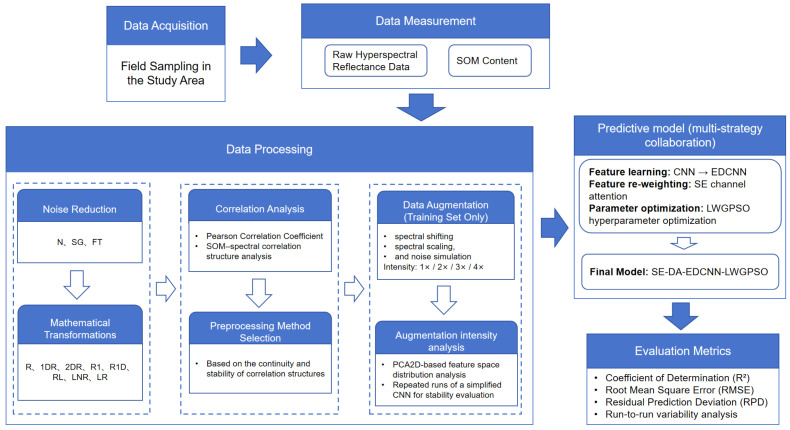
Workflow of the proposed methodology for SOM estimation using hyperspectral data.

Model performance was evaluated using multiple indicators, including the coefficient of determination (R^2^), root mean square error (RMSE), and residual prediction deviation (RPD). The dispersion of results across multiple independent repeated experiments was used to characterize model stability. It should be noted that although the experiments were conducted using laboratory proximal hyperspectral data acquired with an ASD FieldSpec 4 instrument, the proposed framework is primarily intended to analyze the synergistic effects of different modeling strategies under small-sample conditions, and its conclusions are not directly extrapolated to other soil types or remote sensing scales. The overall workflow is illustrated in Figure 2.

### 2.4. Spectral Preprocessing

#### 2.4.1. Savitzky–Golay (SG) Filtering

Savitzky–Golay (SG) filtering smooths the reflectance curve by fitting a local polynomial within a moving window using the least-squares method, thereby suppressing high-frequency noise while preserving spectral absorption features as much as possible:

(1) $$Y_{j}^{*}=\sum_{i=-m}^{m}C_{i}Y_{j+i}$$

where $Y_{j+i}$ denotes the raw spectral reflectance at wavelength *j+i*, $Y_{j}^{*}$ is the smoothed reflectance after Savitzky–Golay filtering, $C_{i}$ represents the smoothing coefficients obtained by least-squares polynomial fitting. In this study, the half-window width was set to m=21, and a third-order polynomial was used for spectral smoothing. This parameter configuration has been widely adopted in soil hyperspectral analysis and is considered to provide an empirical balance between noise suppression and preservation of absorption features [17,18].

#### 2.4.2. Fourier Transform (FT) Filtering

FT filtering decomposes the spectral signal into frequency components, attenuates high-frequency noise, and reconstructs the spectrum via inverse transform. The filtering threshold is:

(2) $$threshold=\alpha \cdot \frac{1}{M}\sum_{k=0}^{M-1}\left|X_{k}\right|$$

where α denotes the threshold coefficient, M represents the number of frequency components, and $X_{k}$ is the amplitude of the k-th frequency component in the Fourier spectrum. In this study, α was set to 0.09, which provided effective suppression of high-frequency noise while retaining the main spectral characteristics, and was therefore adopted consistently for subsequent modeling.

#### 2.4.3. Mathematical Transformations

To investigate the influence of spectral feature engineering on soil organic matter (SOM) prediction under different preprocessing and modeling configurations, this study employed eight commonly used mathematical transformation methods, including raw reflectance (R), first derivative (1DR), second derivative (2DR), reciprocal (R1), first derivative of the reciprocal (R1D), logarithm of the reciprocal (RL), logarithmic transformation (LNR), and reciprocal of the logarithm (LR).

These mathematical transformations aim to adjust spectral representations by reducing baseline effects, enhancing local spectral variations, or emphasizing potential absorption features related to SOM [19]. Their effectiveness is not assumed a priori, but is evaluated through subsequent systematic modeling experiments under different preprocessing combinations and prediction model settings.

### 2.5. Sample Partitioning (SPXY Algorithm)

To ensure representative partitioning of the training and validation sets, the SPXY algorithm was used to jointly maximize spectral and SOM variability [20]:

(3) $$d_{x}\left(p,q\right)=\sqrt{\sum_{j=1}^{N_{b}}{\left(x_{pj}-x_{qj}\right)}^{2}}$$

(4) $$d_{y}\left(p,q\right)=\left|y_{p}-y_{q}\right|$$

(5) $$d_{xy}\left(p,q\right)=\frac{d_{x}\left(p,q\right)}{\underset{p,q\in \Omega }{max}d_{x}\left(p,q\right)}+\frac{d_{y}\left(p,q\right)}{\underset{p,q\in \Omega }{max}d_{y}\left(p,q\right)}$$

where $x_{pj}$ and $x_{qj}$ denote the reflectance values of samples p and q at the j-th wavelength, $N_{b}$ is the number of spectral bands, $y_{p}$ and $y_{q}$ represent the measured SOM contents of samples p and q, respectively, and $d_{x}$ and $d_{y}$ denote the maximum distances used for normalization and Ω represents the set of all samples.

### 2.6. EDCNN Model with Attention and Data Augmentation

#### 2.6.1. Expanded Convolutional Neural Network (EDCNN)

Convolutional neural networks (CNNs) extract features from input spectra through convolution operations and progressively construct feature representations for regression prediction. However, hyperspectral reflectance signals are characterized by strong continuity and absorption features distributed across multiple scales. Traditional CNNs, constrained by fixed receptive fields, have difficulty simultaneously capturing fine-scale local absorption features and spectral correlations over longer wavelength ranges.

**Figure 3:**
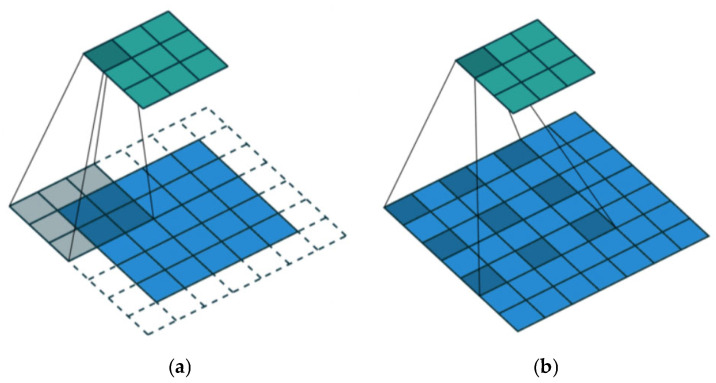
Sampling pattern of an EDCNN convolution kernel at different dilation rates. (**a**) Dilation rate = 1 (standard convolution); (**b**) dilation rate = 2.

Expanded convolutional neural networks (EDCNNs) introduce a dilation rate into the convolutional kernels, thereby enlarging the receptive field without significantly increasing the number of model parameters and enabling joint modeling of spectral features at different scales. This architecture allows more flexible capture of multi-scale absorption patterns and long-range spectral dependencies commonly observed in hyperspectral data, and is particularly suitable for feature learning tasks involving one-dimensional spectral signals. Figure 3 illustrates the sampling patterns of dilated convolution under different dilation rates [21].

#### 2.6.2. Data Augmentation Strategies (DA)

To alleviate the constraints imposed by limited soil sample size on hyperspectral modeling and to enhance model adaptability to spectral perturbations under practical sensing conditions, this study designed three data augmentation strategies to approximately characterize common sensor-related disturbances encountered during hyperspectral acquisition, including baseline shifts, illumination variations, and random noise. All augmentation operations introduce amplitude-controlled local perturbations while preserving the overall structural characteristics of the original spectra, thereby improving the stability and robustness of model training under small-sample conditions [22].

1. Spectral Shifting

Spectral shifting applies a random uniform offset to the entire spectrum to simulate wavelength-independent baseline disturbances caused by sensor calibration errors or response drift. Let the spectral vector be $s=\left[s_{1},s_{2},\ldots ,s_{n}\right],$ where n denotes the number of spectral bands, a random offset $\delta ∼U\left(-0.01,\;0.01\right)$ is added to each spectral band, yielding:

(6) $$s^{\prime }=\left[s_{1}+\delta ,s_{2}+\delta ,\ldots ,s_{n}+\delta \right]$$

This operation introduces slight baseline perturbations while preserving the spectral shape.

2. Spectral Scaling

Spectral scaling applies a random scaling factor to the entire spectrum to simulate the effect of illumination intensity variations on reflectance magnitude. Let the scaling factor be *λ ∼ U* (*0.95, 1.05*) the augmented spectrum is then expressed as:

(7) $$s^{\prime }=\lambda \cdot s$$

This transformation preserves the relative spectral features among bands and only adjusts the overall reflectance amplitude.

3. Spectral Noise Injection

Spectral noise injection adds small random perturbations to each spectral band to approximate common electronic noise and microscale measurement fluctuations during hyperspectral acquisition:

(8) $$s^{\prime }=\left[s_{1}+\epsilon_{1},s_{2}+\epsilon_{2},\ldots ,s_{n}+\epsilon_{n}\right]$$

where $\varepsilon_{i}$ denotes random noise added to the ith spectral band. This strategy introduces controlled spectral variability without disrupting the original spectral structure, thereby helping to improve training stability and prediction reliability under small-sample conditions [23].

#### 2.6.3. Channel Attention Mechanism (SE Module)

**Figure 4:**
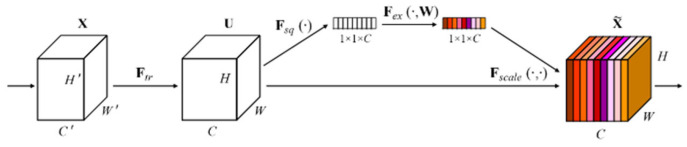
Structure of the SE channel attention module.

The Squeeze-and-Excitation (SE) module adaptively recalibrates feature channels by dynamically adjusting the response strength of different channels, thereby enhancing the effectiveness of feature representations [24]. In hyperspectral modeling tasks, this mechanism helps the network allocate its representational capacity more efficiently under multi-channel feature inputs, enabling the feature learning process to focus more on spectral information that is more beneficial for the prediction task. It should be noted that the channel weights generated by the SE module reflect feature recalibration results under specific data and task conditions, and do not directly correspond to explicit physical absorption mechanisms or the importance of individual spectral bands. A schematic illustration of the SE channel attention module is shown in Figure 4.

#### 2.6.4. Hyperparameter Optimization Using LWGPSO

To reduce the sensitivity of deep models to hyperparameter configurations under small-sample conditions and to mitigate the uncertainty introduced by manual, experience-based tuning, this study employed linear weight particle swarm optimization (LWGPSO) as an automated hyperparameter search method [25]. LWGPSO is built upon the standard particle swarm optimization (PSO) framework and introduces a linearly decreasing inertia weight to balance global exploration and local convergence during the search process, thereby improving the stability of hyperparameter optimization [26].

In this study, LWGPSO was used to systematically search combinations of model hyperparameters under unified validation set evaluation conditions, aiming to reduce the influence of single random initialization or manual tuning on modeling results. The main parameter settings are summarized in Table 1.

#### 2.6.5. SE-EDCNN-DA-LWGPSO Model

This study constructs a comprehensive modeling framework for hyperspectral modeling tasks under small-sample conditions, in which feature extraction, data augmentation, and hyperparameter optimization strategies are applied in a coordinated manner under unified data partitioning and evaluation settings. The model adopts an expanded convolutional neural network (EDCNN) as the backbone architecture. By alternately setting different dilation rates in one-dimensional dilated convolutional layers, spectral features are represented across multiple receptive field scales, thereby enhancing the ability to model complex spectral structures. Data augmentation (DA) strategies are applied only during the training stage to introduce controlled spectral perturbations, improving the stability of the training process under small-sample conditions. During the feature representation stage, an SE channel attention module is incorporated to adaptively recalibrate feature channels. Subsequently, high-dimensional features are mapped to the regression output space through fully connected layers to predict soil organic matter (SOM) content. At the model configuration stage, linear weight particle swarm optimization (LWGPSO) is employed to automatically search for key hyperparameter combinations, reducing the influence of hyperparameter selection on model result stability.

**Figure 5:**
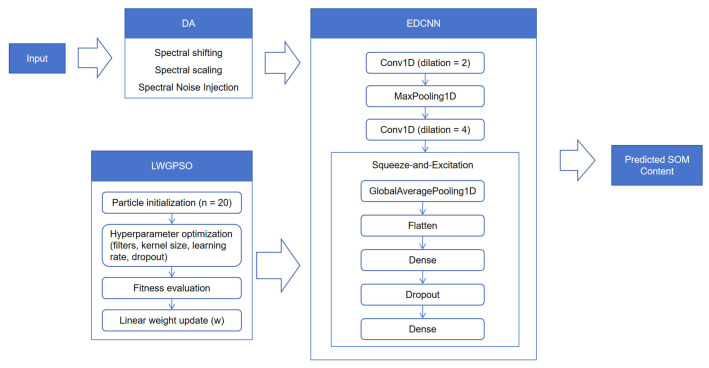
Overall architecture of the SE-EDCNN-DA-LWGPSO model.

These modules jointly operate at the data, feature, and parameter levels to support stable hyperspectral SOM prediction under small-sample conditions. A schematic illustration of the overall architecture is shown in Figure 5.

### 2.7. Other Modeling Methods

#### 2.7.1. Partial Least Squares Regression (PLSR)

PLSR is a linear regression approach that projects high-dimensional spectral data into a latent low-dimensional space while maximizing covariance with SOM. It is widely used due to its stability in handling multicollinearity [27].

#### 2.7.2. Random Forest (RF)

RF is a nonlinear ensemble learning algorithm that constructs multiple decision trees using random feature and sample subsets [28].

It reduces overfitting risk and can identify important spectral wavelengths.

#### 2.7.3. Support Vector Regression (SVR)

SVR introduces slack variables and a penalty term to balance model complexity and fitting accuracy. It effectively models nonlinear relationships between spectral features and SOM and is robust under small-sample conditions [29].

### 2.8. Evaluation Metrics

To enable an objective comparison of predictive performance across different modeling approaches under a unified data partitioning and validation scheme, three commonly used evaluation metrics were adopted: the coefficient of determination (R^2^), the root mean square error (RMSE), and the ratio of performance to deviation (RPD). These metrics were jointly used to assess the performance of both traditional machine learning models and deep learning models in soil organic matter (SOM) prediction.

The coefficient of determination (R^2^) quantifies the proportion of variance in the measured SOM values that is explained by the model predictions. An R^2^ value closer to 1 indicates better agreement between predicted and measured values. It is defined as:

(9) $$R^{2}=1-\frac{\sum_{i=1}^{n}{\left(y_{i}-{\hat{y}}_{i}\right)}^{2}}{\sum_{i=1}^{n}{\left(y_{i}-\overset{¯}{y}\right)}^{2}}$$

where $y_{i}$ is the measured SOM value, ${\hat{y}}_{i}$ is the predicted value, $\overset{¯}{y}$ is the mean of measured values, and n is the number of samples.

The root mean square error (RMSE) measures the absolute deviation between predicted and observed SOM values. A lower RMSE indicates higher prediction accuracy. It is calculated as:

(10) $$\mathrm{RMSE}=\sqrt{\frac{1}{n}\sum_{i=1}^{n}{\left(y_{i}-{\hat{y}}_{i}\right)}^{2}}$$

The ratio of performance to deviation (RPD) evaluates model performance by comparing the standard deviation (SD) of measured SOM values with the RMSE. A larger RPD indicates better model predictive performance. The metric is defined as:

(11) $$RPD=\frac{SD}{RMSE}$$

## 3. Results

### 3.1. Characteristics of SOM Content and Raw Spectrum

A total of 278 soil samples were collected in this study, and the descriptive statistics of their soil organic matter (SOM) content are summarized in Table 2. Overall, the SOM content of the samples exhibits a moderate to relatively high degree of variability, while the sample size remains limited. Under such conditions, the mapping relationship between soil spectral features and SOM content is generally complex. Model prediction results are influenced not only by the modeling approach but also by the distribution pattern of samples in the spectral feature space [30].

Therefore, prior to subsequent model construction and performance evaluation, it is necessary to analyze the overall distribution characteristics of the samples, providing a basis for subsequent sample partitioning strategies and modeling stability assessment.

**Figure 6:**
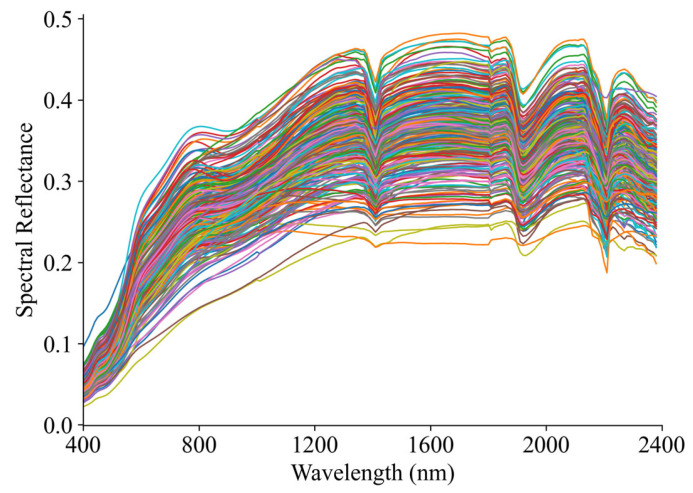
Raw reflectance spectra of soil samples.

Figure 6 illustrates the raw reflectance spectra of soil samples over the 400–2400 nm wavelength range. Overall, the spectral curves of different samples exhibit similar general shapes, while notable differences are observed in reflectance magnitude. In the visible to near-infrared region, reflectance shows a typical continuous variation with wavelength, whereas several distinct absorption features can be observed in the shortwave infrared region. The primary differences among samples are mainly reflected in reflectance magnitude rather than in overall spectral shape. Samples with higher SOM content generally exhibit lower overall reflectance levels, whereas samples with lower SOM content show higher reflectance. These differences indicate that, in subsequent modeling, it is necessary to apply appropriate data representation and feature processing strategies to achieve a unified and stabilized expression of spectral information, thereby providing reliable inputs for modeling analysis under small-sample conditions [31].

### 3.2. Correlation Analysis of Different Preprocessing Methods

**Figure 7:**
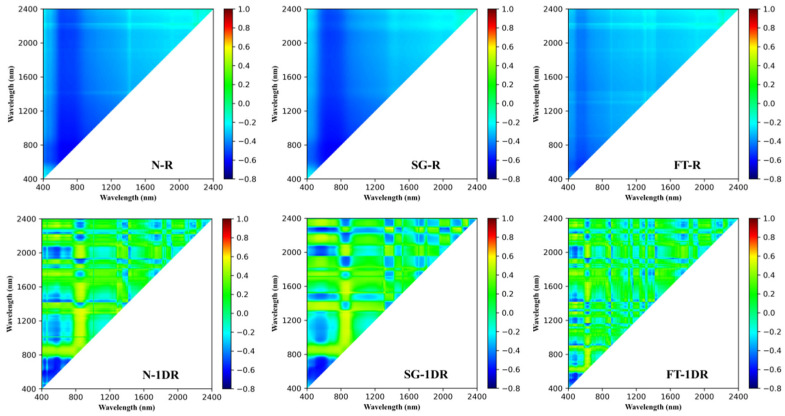

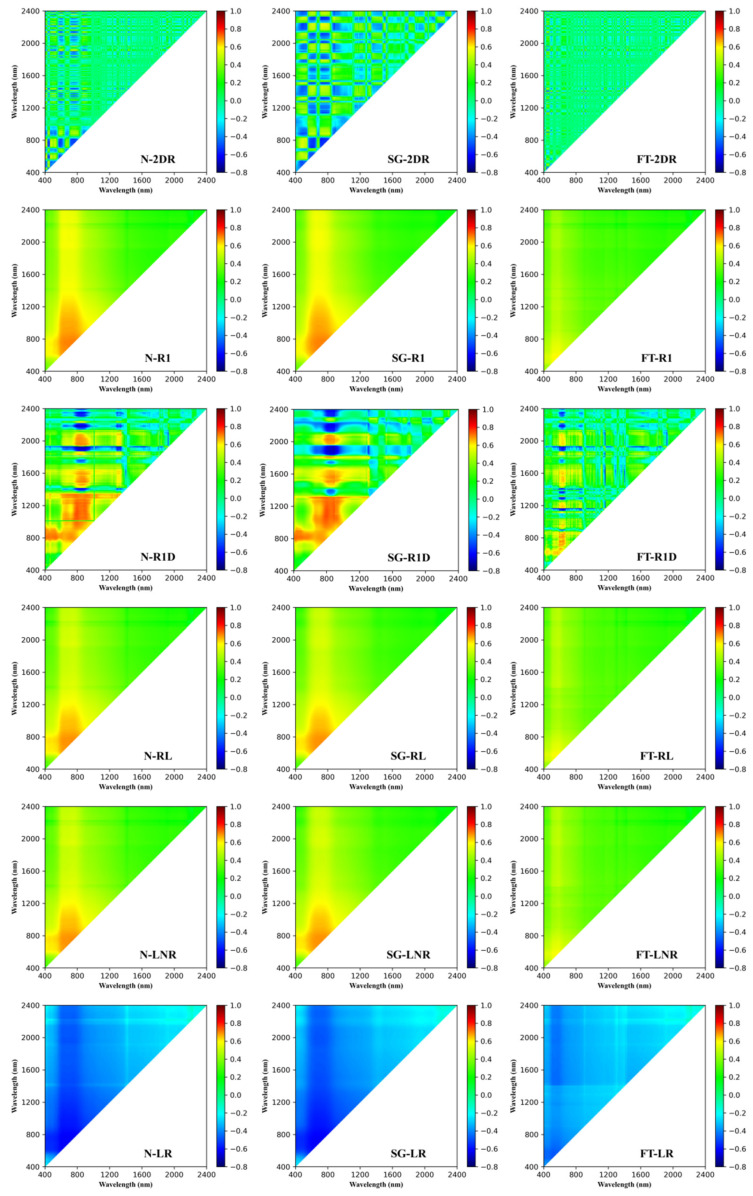
Pearson correlation heatmap of SOM and spectral bands under different preprocessing methods.

To systematically evaluate the influence of different spectral preprocessing methods on the relationship between reflectance and soil organic matter (SOM) content, this study constructed 24 preprocessing combinations, including three denoising strategies (no denoising, N; Savitzky–Golay filtering, SG; Fourier transform filtering, FT) and eight mathematical transformation forms (R, 1DR, 2DR, R1, R1D, RL, LNR, and LR). In this study, the Pearson correlation coefficient was used to quantitatively characterize the degree of correlation between individual spectral bands and SOM content [32], based on which correlation heatmaps were constructed (Figure 7) to compare the distribution characteristics of SOM–spectral correlation structures under different preprocessing combinations.

As shown in Figure 7, different preprocessing combinations exhibit noticeable differences in terms of the spatial continuity and local contrast of correlation structures. Some mathematical transformations (e.g., R, LR, LNR, RL, and R1) produce relatively smooth correlation distributions with limited local variation, making it difficult to clearly distinguish differences in correlation structures among spectral bands. In contrast, although 2DR enhances local correlation variations in certain regions, its correlation structure exhibits pronounced high-frequency fragmentation and poor continuity, which may amplify noise-related components. By comparison, both 1DR and R1D show relatively clear correlation structures under different denoising strategies (N, SG, and FT). Specifically, 1DR maintains a relatively continuous and balanced correlation distribution over a wide wavelength range, whereas R1D exhibits more concentrated high-correlation structures in certain spectral regions. These differences reflect the distinct emphases of different mathematical transformations in adjusting spectral correlation structures.

Based on these characteristics, 1DR and R1D were selected as representative mathematical transformation forms for subsequent modeling experiments. It should be emphasized that this selection process was based solely on the distribution characteristics and continuity of the correlation structures, without involving any model training results or prediction performance metrics, in order to avoid post hoc selection bias.

To quantitatively describe the correlation structure differences shown in Figure 7, several statistical indicators were further calculated, including the mean absolute correlation coefficient (Mean |corr|), the proportion of correlation coefficients with absolute values greater than 0.6 (Ratio (|r| > 0.6)), the top 1% absolute correlation coefficients (Top 1% |corr|), and the 95th percentile of absolute correlation coefficients (P95 |corr|). The corresponding results are summarized in Table 3, illustrating the differences in correlation structure characteristics between 1DR and R1D under SG and FT denoising conditions.

As shown in Table 3, 1DR exhibits higher values of Mean |corr| overall, indicating a more balanced correlation structure across the full spectral range. In contrast, R1D shows more pronounced performance in terms of the proportion of high-correlation regions (Ratio (|r| > 0.6)) and high-quantile correlation indicators, reflecting the formation of more concentrated correlation structures within specific spectral regions. These quantitative results further complement the correlation structure differences revealed in Figure 7.

Based on the above correlation structure analysis, 1DR and R1D were incorporated into the modeling experiments to examine the roles of their respective correlation structure characteristics in practical SOM prediction tasks. The prediction results under different preprocessing combinations and modeling methods are presented in Table 4.

The results show that the predictive performance of the linear model PLSR is generally lower than that of the nonlinear models RF and SVR, which is consistent with previous studies indicating that nonlinear algorithms have advantages in characterizing complex relationships in hyperspectral data [33]. Among different denoising and modeling combinations, RF-based models exhibit overall superior performance, with both SG–1DR–RF and FT–1DR–RF achieving relatively high prediction accuracy. For SG–1DR–RF, the R^2^, RMSE, and RPD values on the training set are 0.904, 2.520 g·kg^−1^, and 3.223, respectively, while the corresponding values on the validation set are 0.865, 2.981 g·kg^−1^, and 2.725, indicating relatively stable predictive performance. In comparison, FT–1DR–RF achieves a slightly higher R^2^ on the validation set (0.871), with RMSE and RPD values close to those of SG–1DR–RF, suggesting that the overall predictive capability of the 1DR–RF combination is similar under different denoising strategies.

Considering both training and validation results, SG–1DR–RF does not exhibit a pronounced performance imbalance, and the SG–1DR preprocessing maintains relatively consistent performance across the PLSR, RF, and SVR models, demonstrating good methodological adaptability [34]. It should be noted that the above comparison is based on single-run experimental results and is mainly intended to analyze the relative performance of different preprocessing combinations under the current dataset conditions.

### 3.3. Effects of Data Augmentation on Sample Distribution and Model Stability

Considering that SG–1DR exhibits more consistent performance across different models, spectra preprocessed with SG–1DR were uniformly adopted as the base input for subsequent modeling and data augmentation. Under the premise of maintaining spectral physical consistency, introducing controlled data augmentation to moderately supplement the original sample distribution is of significant importance for improving training stability and robustness under small-sample conditions [35]. It should be noted that higher data augmentation intensity is not necessarily better, as excessive augmentation may introduce too many boundary samples in the statistical sense, thereby adversely affecting model generalization performance.

#### 3.3.1. Data Augmentation Design and Sample Partitioning

To ensure consistency in experimental partitioning, data augmentation was applied only to the training set after SPXY-based sample partitioning, while the validation set remained unchanged throughout all experiments. Augmentation operations were performed in the spectral space after SG–1DR preprocessing. For each training sample, augmented samples were generated at a predefined factor k (k = 1~4) using controlled perturbations, including spectral shifting, global scaling disturbances, and additive noise injection. All perturbations were constrained within small amplitude ranges to ensure that the augmented samples remained consistent with the original samples in terms of spectral shape and physical meaning. In this study, five augmentation levels ranging from 0× to 4× were considered, and the training sample size was progressively expanded while keeping the validation set fixed. The composition of training and validation sample numbers under different augmentation factors is presented in Table 5.

In this study, principal component analysis (PCA) was applied to reduce the dimensionality of the high-dimensional spectral features after SG–1DR preprocessing, and the PCA model was fitted using the original training samples. Subsequently, both the original samples and their corresponding augmented samples were jointly projected into a two-dimensional feature space defined by the first two principal components (PCA2D), in order to analyze the overall distributional changes of training samples in feature space under different data augmentation factors [36]. This analysis aims to examine whether data augmentation introduces significant distributional drift or structural distortion.

**Figure 8:**
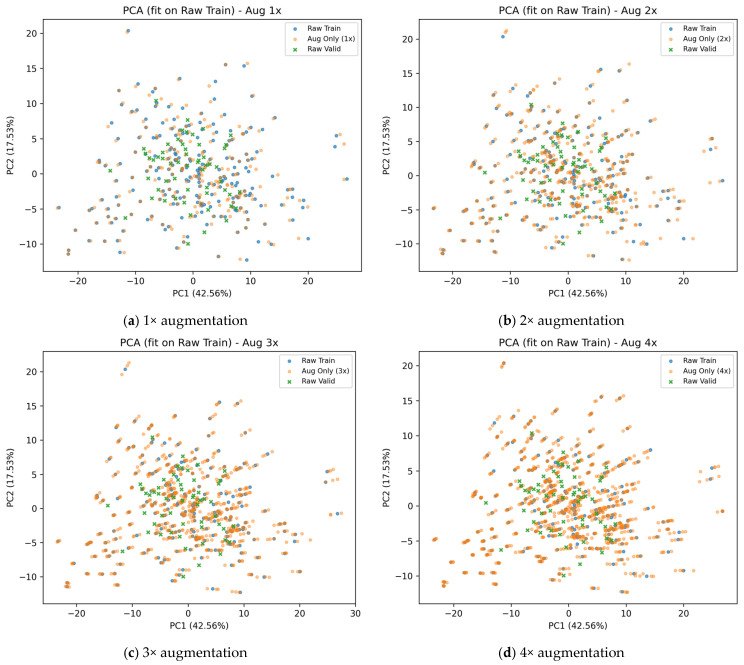
PCA2D visualization of training sample distributions under different data augmentation factors.

Within the PCA2D space, several statistical indicators were calculated for training samples under different augmentation factors, including the mean distance, distance standard deviation, coefficient of variation, centroid shift, and coverage radius ratio. These metrics were used to quantitatively characterize the structure of sample distributions. The corresponding results are summarized in Table 6 and visualized in Figure 8.

The results indicate that under different data augmentation factors, the augmented samples are still distributed around the original training sample region, without exhibiting evident centroid shifts or the formation of new independent cluster structures. Across all augmentation levels, the variations in mean distance, distance standard deviation, and coefficient of variation are relatively small; the centroid shift remains close to zero, and the coverage radius ratio is close to one. These findings suggest that data augmentation does not significantly alter the overall distribution structure of training samples in the low-dimensional feature space. Further comparison shows that the 1× augmented samples highly overlap with the non-augmented samples in feature space, whereas higher augmentation factors (e.g., 4×) are mainly reflected by increased sample density near the original distribution boundaries rather than by the emergence of new representative structures. Considering both the incremental distributional information and structural stability, moderate augmentation factors (2×–3×) achieve a balance by maintaining distribution continuity and consistency while moderately densifying the local feature space. The above analysis is intended solely to characterize sample distribution properties.

#### 3.3.2. Performance Evaluation of Augmented Samples Using CNN

To avoid interference from complex model architectures in result interpretation and to highlight the effect of data augmentation intensity itself, a convolutional neural network (CNN) with a simple structure was selected as the reference model. This model contains only basic one-dimensional convolution, pooling, and fully connected layers, without incorporating dilated convolutions, attention mechanisms, or hyperparameter optimization, and serves as a low-complexity baseline for analyzing prediction performance variability under different augmentation conditions.

Under identical network architecture and training strategies, five independent repeated experiments were conducted for the selected augmentation factors (0×, 2×, and 3×), and model performance was evaluated on the same validation set to reduce the influence of random factors on result analysis. The corresponding run-level validation results are presented in Table 7.

**Figure 9:**
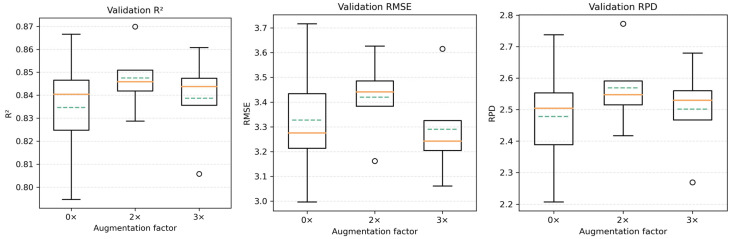
Boxplot distributions of CNN validation R^2^, RMSE, and RPD across different data augmentation factors.

Under the no data augmentation (0×) condition, the CNN model exhibits pronounced variability in validation performance across different runs, with wide ranges of R^2^, RMSE, and RPD values, indicating that the model is highly sensitive to training process perturbations under small-sample conditions. After introducing 2× data augmentation, the prediction results from independent runs show a more concentrated distribution: extreme low R^2^ values no longer occur, and the variability of RMSE and RPD is substantially reduced, reflecting an improvement in training stability. In contrast, under the 3× augmentation condition, although some runs still achieve relatively favorable prediction performance, the performance differences among runs increase again, and performance degradation is observed in certain runs. This suggests that under stronger augmentation intensity, the consistency of model results decreases. To visually illustrate the overall variability of model prediction performance across multiple runs, boxplot visualizations of validation R^2^, RMSE, and RPD are presented in Figure 9.

As shown in Figure 9, compared with the larger interquartile ranges under the 0× condition, the box ranges of all evaluation metrics under the 2× augmentation condition exhibit a clear convergence, whereas the distributions under the 3× condition show an expanding trend again.

Based on the run-level results, the mean values and standard deviations of validation R^2^ and RMSE under different augmentation factors were calculated (Table 8). The results indicate that both metrics achieve their lowest standard deviations under the 2× data augmentation condition, while the average predictive performance of the model does not exhibit a significant change.

Based on the above discussion, 2× data augmentation achieves a relatively balanced trade-off between sample distribution rationality and model training stability. Therefore, all subsequent model construction and accuracy comparison experiments were conducted based on the 2× data augmentation strategy.

### 3.4. Final Model Performance and Ablation Analysis

In Section 3.3, a simplified CNN model was used to systematically analyze the influence of different data augmentation factors on training stability under small-sample conditions, based on which a 2× data augmentation strategy was selected for subsequent experiments. Building on this basis, this section compares the predictive performance of the final proposed model and its structural variants, and quantitatively evaluates the contributions of key modules to performance improvement and stability enhancement through ablation experiments.

#### 3.4.1. Comparative Analysis of Predictive Performance Across Different Models

Under unified data partitioning, training procedures, and evaluation metric settings, ten independent repeated experiments were conducted for the CNN, EDCNN, and their improved variants obtained through stepwise ablation within the final modeling framework. The mean values and standard deviations of predictive performance on the validation set for each model are summarized in Table 9, with CNN serving as the baseline model for the ablation experiments.

As shown in Table 9, significant differences in predictive performance are observed among the models on the validation set. Overall, with the progressive introduction of model structures and training strategies, R^2^ increases continuously, RMSE decreases gradually, and RPD is markedly improved. Compared with the baseline CNN, the introduction of dilated convolutions in EDCNN results in a clear improvement in predictive performance, highlighting the importance of multi-scale feature modeling in SOM spectral inversion. On this basis, further incorporating data augmentation and the SE channel attention mechanism leads to additional performance gains, with SE-EDCNN-DA exhibiting superior overall predictive capability. With the further introduction of LWGPSO, the model not only maintains high average predictive accuracy but also shows a further reduction in the dispersion of results across multiple independent runs, indicating improved training consistency.

**Figure 10:**
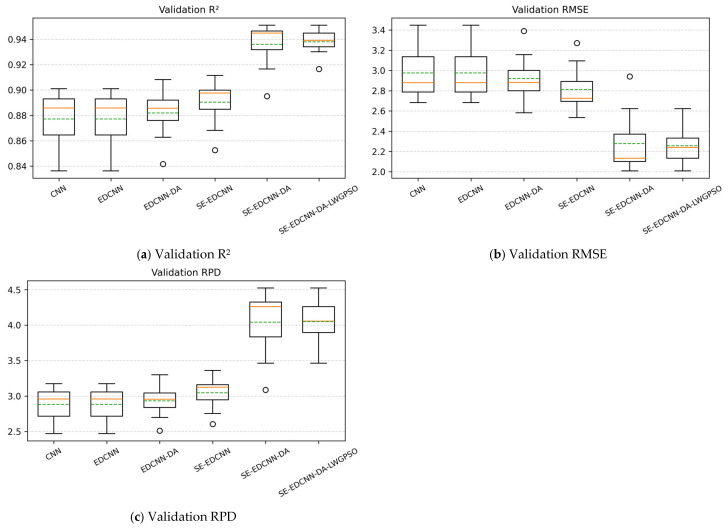
Boxplot distributions of validation R^2^, RMSE, and RPD for different models across 10 independent runs. Orange solid and green dashed lines denote the median and mean, respectively.

Based on the ten run-level validation results corresponding to Table 9, boxplots of R^2^, RMSE, and RPD for each model were generated, as shown in Figure 10.

It can be observed that, as the model architecture is progressively refined, the boxplot distributions of all evaluation metrics exhibit an overall trend of convergence toward the median. Compared with EDCNN, EDCNN-DA, and SE-EDCNN, both SE-EDCNN-DA and SE-EDCNN-DA-LWGPSO show markedly narrower distribution ranges across all three metrics, with high consistency between mean and median values and only a small number of outliers appearing in a few runs.

#### 3.4.2. Ablation Analysis of Key Model Modules

**Figure 11:**
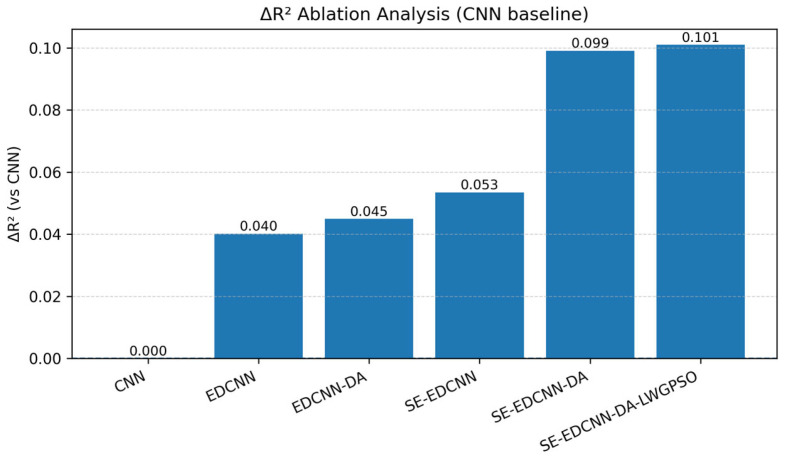
ΔR^2^-based ablation analysis of different model variants relative to the CNN baseline.

To quantitatively evaluate the contribution of each key module to model predictive performance, a series of ablation models were constructed within the final modeling framework by progressively removing individual functional modules. The basic CNN, which does not include dilated convolutions, the SE attention mechanism, or data augmentation strategies, was used as the baseline model for the ablation analysis. Based on the mean validation R^2^ values obtained from ten independent runs for each model in Table 9, the performance gains of different models relative to the baseline CNN were calculated and visualized in terms of ΔR^2^ (Figure 11).

As shown in Figure 11, compared with the baseline CNN (R^2^ = 0.837), the introduction of dilated convolutional structures increases the validation R^2^ of EDCNN to 0.877 (ΔR^2^ ≈ 0.040), indicating that multi-scale feature modeling plays a significant role in promoting SOM spectral inversion. On this basis, incorporating data augmentation (EDCNN-DA) further improves R^2^ to 0.882 (ΔR^2^ ≈ 0.045); however, the gain relative to EDCNN is limited, suggesting that in this study data augmentation mainly serves as an auxiliary strategy to alleviate training instability under small-sample conditions rather than directly yielding a substantial accuracy improvement.

In contrast, introducing the SE channel attention mechanism (SE-EDCNN) increases the validation R^2^ to 0.890 (ΔR^2^ ≈ 0.053), with a larger performance gain than that achieved by data augmentation alone. This indicates that the attention mechanism can further strengthen the representation of spectral features highly correlated with SOM prediction on the basis of multi-scale feature extraction. When data augmentation and the SE attention mechanism are jointly incorporated (SE-EDCNN-DA), the validation R^2^ increases markedly to 0.936 (ΔR^2^ ≈ 0.10), demonstrating a clear synergistic gain. After further optimizing key model hyperparameters (SE-EDCNN-DA-LWGPSO), the validation R^2^ reaches 0.938. Although the improvement is modest, the results from multiple independent runs indicate that LWGPSO helps reduce performance fluctuations under different random initialization conditions, thereby enhancing training consistency and stability.

#### 3.4.3. Final Model Analysis

Although Table 9 provides a statistical comparison of the average predictive performance of different models, this section further evaluates the stability and result consistency of SE-EDCNN-DA-LWGPSO from a run-level perspective under multiple independent runs, in order to verify whether its predictive performance is dominated by individual runs.

Table 10 summarizes the prediction results of this model on the training and validation sets across ten independent runs.

It can be observed that the validation R^2^, RMSE, and RPD values across different runs are distributed within relatively narrow ranges, with no evident performance degradation or anomalous runs, indicating good model robustness. The performance gaps between the training and validation sets remain within reasonable bounds, and no systematic overfitting tendency is observed. Combined with the validation performance distributions of the final model shown in Figure 10, it can be concluded that the model exhibits good stability and consistency under multiple independent runs, and its predictive performance is not driven by a single random experiment.

## 4. Discussion

In hyperspectral soil organic matter (SOM) prediction tasks under small-sample conditions, model predictions are often highly sensitive to data partitioning, parameter initialization, and modeling configurations, leading to pronounced performance fluctuations across independent training processes. Such variability limits the stability and reproducibility of modeling results. Against this background, the focus of this study is not the optimal predictive accuracy under a single training setting, but rather the mechanisms by which different modeling strategies influence prediction stability and consistency under small-sample conditions.

The experimental results indicate that the proposed multi-strategy collaborative framework exhibits clear advantages in reducing result variability. In this study, data augmentation primarily serves as a training process regulation strategy. By locally densifying the original sample distribution without introducing significant distributional drift, data augmentation effectively reduces model sensitivity to random perturbations, thereby decreasing the dispersion of prediction results across independent runs. This finding suggests that, in small-sample scenarios, the role of data augmentation is manifested more in stability enhancement than in introducing new discriminative information through feature space expansion.

At the model structure level, the multi-scale expanded convolutional neural network (EDCNN) enhances the capability to model complex spectral patterns by incorporating feature extraction paths with different receptive field scales, providing a more robust feature representation basis for stable prediction. On this basis, the SE channel attention mechanism further adaptively reweights intermediate feature responses, helping to mitigate the interference of redundant features during training. It should be emphasized that the SE mechanism is not used in this study for band-level or physically interpretable analysis; its role is mainly reflected in improving feature utilization efficiency and training consistency. The function of linear weight generalized particle swarm optimization (LWGPSO) in this study is to identify hyperparameter configurations with relatively stable predictive performance, thereby further reducing the influence of manual experience and random initialization on modeling results. This strategy enhances reproducibility at the model configuration level and complements data augmentation and architectural design.

Overall, improvements in average predictive performance are mainly attributed to enhanced representational capacity brought by multi-scale feature extraction and attention mechanisms, whereas data augmentation and hyperparameter optimization strategies primarily contribute to reducing the dispersion of results across independent training processes. These strategies exhibit synergistic effects in this study, jointly promoting improved stability and consistency of model predictions under small-sample conditions.

It should be noted that the discussions and conclusions of this study are confined to the investigated data conditions and application scenario. The analyses are primarily intended to facilitate understanding of how different modeling strategies influence model behavior and stability in small-sample hyperspectral regression tasks.

## 5. Conclusions

This study addresses the issue of performance variability in hyperspectral soil organic matter (SOM) prediction under small-sample conditions across different modeling configurations and repeated training processes, and constructs and evaluates a multi-strategy collaborative deep learning framework (SE-EDCNN-DA-LWGPSO). Unlike traditional modeling approaches that focus on achieving optimal predictive accuracy in a single run, this study emphasizes the stability and consistency of model predictions, and systematically analyzes the effects of different strategies through multiple independent repeated experiments.

The experimental results demonstrate that the multi-scale dilated convolutional structure and the SE channel attention mechanism provide a more robust feature representation basis for the model, contributing to improvements in average predictive performance. Meanwhile, data augmentation strategies based on spectral perturbations and the linear weight generalized particle swarm optimization method mainly act at the training process and parameter configuration levels, effectively reducing the dispersion of prediction results across independent runs. These strategies exhibit synergistic effects in this study, jointly promoting enhanced stability and consistency of model predictions under small-sample conditions.

Further analysis reveals that the overall improvement in model performance does not result from a simple accumulation of individual strategies, but rather from the coordinated interaction between enhanced structural modeling capability and stability regulation mechanisms. This finding indicates that, for hyperspectral regression tasks with limited sample sizes, jointly considering model architecture design, data perturbation strategies, and parameter search methods is of great importance for improving the reliability of modeling results.

It should be noted that the conclusions of this study are based on hyperspectral data acquired under laboratory conditions from a single region and a single soil type, and their applicability is therefore mainly confined to small-sample modeling scenarios under similar data conditions. Although the proposed multi-strategy collaborative modeling concept has certain methodological reference value, its specific performance remains dependent on data characteristics, target variables, and application scenarios. Future work will further validate its applicability under more diverse data conditions.

## Figures and Tables

**Table 1:** Core parameters of the LWGPSO particle swarm.

Parameter	Value
Particle number	20
Max iterations	50
Learning factor 1	2
Learning factor 2	2
Max inertia weight	0.9
Min inertia weight	0.4

**Table 2:** Statistical characteristics of SOM content in soil samples.

Sample Type	Count	Max (g·kg^−1^)	Min (g·kg^−1^)	Mean (g·kg^−1^)	SD (g·kg^−1^)	CV (%)
Total samples	278	64.53	6.66	22.31	8.14	36.47

**Table 3:** Quantitative statistics of SOM–spectral interaction characteristics under selected preprocessing methods.

Denoising Method	Mathematical Transformation	Mean |corr|	Ratio (|r| > 0.6)	Top 1% |corr|	P95 |corr|
SG	1DR	0.53	0.13	0.80	0.74
	R1D	0.44	0.20	0.84	0.79
FT	1DR	0.45	0.03	0.66	0.58
	R1D	0.32	0.06	0.76	0.65

**Table 4:** Prediction accuracy of SOM under different preprocessing methods.

Denoising Method	Mathematical Transformation	Model	Training			Validation		
			R^2^	RMSE	RPD	R^2^	RMSE	RPD
SG	1DR	PLSR	0.638	4.888	1.662	0.600	5.138	1.581
		RF	0.904	2.520	3.223	0.865	2.981	2.725
		SVR	0.706	4.723	1.845	0.647	3.053	1.683
	R1D	PLSR	0.696	4.528	1.815	0.566	5.087	1.519
		RF	0.913	2.425	3.388	0.815	3.319	2.328
		SVR	0.883	2.980	2.923	0.640	3.041	1.668
FT	1DR	PLSR	0.647	4.842	1.684	0.605	5.026	1.592
		RF	0.887	2.741	2.974	0.871	2.874	2.738
		SVR	0.791	3.986	2.190	0.704	2.749	1.838
	R1D	PLSR	0.708	4.404	1.850	0.613	4.998	1.607
		RF	0.866	2.982	2.732	0.850	3.109	2.583
		SVR	0.792	3.977	2.195	0.543	3.296	1.480

**Table 5:** Sample composition under different augmentation factors.

Aug Factor	Original Train (n)	Aug-Only (n)	Total Train (n)	Validation (n)
0×	223	0	223	55
1×	223	223	446	55
2×	223	446	669	55
3×	223	669	892	55
4×	223	892	1115	55

**Table 6:** Quantitative comparison of sample distribution characteristics under different augmentation factors.

Aug Factor	Mean Distance	Std. Distance	CV Std	Centroid Shift	Radius Ratio
0×	9.402	5.583	0.594	0.000	1.000
1×	9.416	5.578	0.592	0.025	1.000
2×	9.426	5.568	0.591	0.009	1.000
3×	9.401	5.598	0.595	0.002	1.026
4×	9.411	5.558	0.591	0.009	1.001

**Table 7:** Run-level validation results of the CNN model under different data augmentation factors.

Aug Factor	Run	Validation		
		R^2^	RMSE	RPD
0×	5	0.8466	3.2133	2.5530
		0.8405	3.2759	2.5042
		0.7947	3.7171	2.2070
		0.8666	2.9966	2.7376
		0.8248	3.4341	2.3888
2×	5	0.8288	3.6270	2.4171
		0.8510	3.3837	2.5909
		0.8699	3.1619	2.7727
		0.8419	3.4854	2.5153
		0.8459	3.4414	2.5475
3×	5	0.8608	3.0612	2.6799
		0.8058	3.6153	2.2691
		0.8357	3.3255	2.4669
		0.8474	3.2047	2.5598
		0.8438	3.2425	2.5300

Note: The CNN refers to a simplified network architecture.

**Table 8:** Summary statistics of CNN validation performance under different augmentation factors.

Aug Factor	Mean R^2^	Std R^2^	Mean RMSE	Std RMSE
0×	0.835	0.027	3.327	0.268
2×	0.848	0.016	3.420	0.168
3×	0.839	0.020	3.290	0.197

**Table 9:** Prediction accuracy of different modeling approaches for SOM estimation.

Model	Run	Validation		
		R^2^	RMSE	RPD
CNN	10	0.837 ± 0.023	3.305 ± 0.224	2.492 ± 0.163
EDCNN	10	0.877 ± 0.022	2.976 ± 0.263	2.883 ± 0.243
EDCNN-DA	10	0.882 ± 0.019	2.920 ± 0.231	2.935 ± 0.225
SE-EDCNN	10	0.890 ± 0.018	2.812 ± 0.223	3.047 ± 0.228
SE-EDCNN-DA	10	0.936 ± 0.018	2.279 ± 0.298	4.042 ± 0.462
SE-EDCNN-DA-LWGPSO	10	0.938 ± 0.010	2.256 ± 0.176	4.050 ± 0.305

Note: The CNN serves as the baseline model for the ablation experiments.

**Table 10:** Training and validation results of the SE-EDCNN-DA-LWGPSO model across 10 independent runs.

Run	Training			Validation		
	R^2^	RMSE	RPD	R^2^	RMSE	RPD
1	0.9743	1.2593	6.2418	0.9166	2.6234	3.4636
2	0.9803	1.1031	7.1259	0.9330	2.3525	3.8626
3	0.9846	0.9761	8.053	0.9511	2.0085	4.5242
4	0.9829	1.027	7.6541	0.9467	2.0976	4.332
5	0.9825	1.0407	7.5529	0.9408	2.2117	4.1084
6	0.9828	1.0312	7.6227	0.9462	2.1076	4.3113
7	0.9788	1.1447	6.8666	0.9374	2.2736	3.9967
8	0.9793	1.1306	6.9527	0.9301	2.4022	3.7826
9	0.981	1.0841	7.2508	0.9376	2.2694	4.0039
10	0.9804	1.0992	7.151	0.9408	2.2105	4.1107

## Data Availability

The data used in this study are not publicly available as they are internal datasets of the research group. The data may be made available from the corresponding author upon reasonable request.

## Abbreviations

The following abbreviations are used in this manuscript:

SOM	Soil Organic Matter
Vis–NIR	Visible and Near-Infrared
CNN	Convolutional Neural Network
EDCNN	Expanded (Dilated) Convolutional Neural Network
SE	Squeeze-and-Excitation
DA	Data Augmentation
LWGPSO	Linearly Weighted Generalized Particle Swarm Optimization
RMSE	Root Mean Square Error
RPD	Ratio of Performance to Deviation
R^2^	Coefficient of Determination
